# A new approach for investigation of person–environment interaction effects in research involving health outcomes

**DOI:** 10.1007/s10433-018-0480-5

**Published:** 2018-06-12

**Authors:** Björn Slaug, Susanne Iwarsson, Jonas Björk

**Affiliations:** 1 Department of Health Sciences, Faculty of Medicine, Lund University, Box 157, 221 00 Lund, Sweden; 2 Department of Occupational and Environmental Medicine, Lund University, Lund, Sweden

**Keywords:** Accessibility, Causal relations, Ecological Theory of Ageing, Housing Enabler, Multi-variable analyses

## Abstract

Recurrent use of the Housing Enabler instrument has highlighted methodological challenges of broader scientific interest, namely interactions between personal functional capacity (*P*) and exposures to features (here potential barriers) in the built housing environment (*E*). This study aimed to propose and illustrate an analytic approach, separating *P *× *E* interaction effects (here accessibility problems) from main effects of *P* and *E*, in studies where *P* and *P *× *E* are strongly interrelated. Four datasets representing different populations of older people in the context of housing were used. The datasets (*N* = 1910) comprised data on *P*, *E* and *P* × *E* interactions as well as health-related variables. A two-step analytic procedure was performed: (1) a measure of environmental barriers net of functional capacity was obtained from residuals of linear regression analysis between P (independent) and *P* × *E* (dependent); (2) logistic regression analyses with self-rated general health and I-ADL, respectively, as dependent variables to explore interaction effects using the *P* × *E* residuals from the previous step. The association between *P* and *P* × *E* was similar across the four datasets (*r *≥ 0.80,  *p* < 0.001). In the logistic regression analyses, including *P*, both categorized and continuous *P* × *E* residuals were clearly associated with self-rated general health (*p *< 0.001 and *p *= 0.026), whereas the associations with I-ADL were less consistent (*p *= 0.275 and *p* = 0.002, respectively). The new two-step—instead of single-step—analytic approach proposed for investigating *P* × *E* interaction effects in studies involving health outcomes emerged as promising. The new approach has the potential of increasing the possibilities to adequately represent theoretical concepts and assumptions and rigorously test their effects.

## Introduction

The identification and specification of associations between explanatory and outcome variables is at the core of several research fields within the health, behavioural and social sciences and of great relevance for ageing research. Given the complex dynamics often targeted, attention is increasingly focused on so-called interaction effects, that is, associations where the influence of explanatory variables is dependent on moderating factors. This increased attention has resulted in numerous treatments in the recent literature of various design, analysis and interpretation considerations when examining such associations between explanatory and outcome variables (see, e.g. Hayes and Rockwood 2016; Kraemer 2016; Magill 2011).

The study of interaction effects can be described as a way of addressing research questions that ask “when” and “under what conditions” certain kinds of such effects occur. This can enrich our understanding and help to explain why otherwise known relationships between two variables vary in different contexts. A recent example from ageing research is a study that examined the relationship between cognitive performance and well-being in a sample of older adults and found this relationship to be moderated by sensory impairments (Wettstein et al. 2015). The notion of interaction is in a general sense dependent on the scale with which the exposure effect is assessed, and it is often argued in epidemiology that interaction should be defined as a departure from additivity of risks (Rothman et al. 2008). The standard single-step approach in statistical interaction analysis is to establish a multi-variable regression model where the main effects of single variables of interest are included simultaneously together with a cross-product term. The *p* value for the cross-product term summarizes the overall empirical evidence for the existence of interaction between the factors under study (Jaccard and Turrisi 2003). However, for theoretical models assuming that a health outcome is dependent on phenomena that both have main and interaction effects it is challenging to find the optimal statistical procedures that are scientifically sound; that is, that the theoretical concepts are adequately represented in the statistical procedure and that the effects predicted by the model are possible to test.

In ageing research, the Ecological Theory of Ageing (Lawton and Nahemow 1973) is one of the most cited explanatory models. It is frequently used in studies analysing health-related outcomes of the interaction between the capacity of the person and the demands of the environment (see, e.g. Wahl and Weisman 2003; Wahl et al. 2012), where the interaction often has been labelled person–environment fit. In the current study, we have therefore chosen an application of the Ecological Theory of Ageing as a study case for statistical analysis and interpretation of interaction effects. An important aspect of the Ecological Theory of Ageing is the docility hypothesis (Lawton 1986), according to which a balance between the person’s capacity and environmental press can be achieved by changing one or the other component, or both. Hence, even if the person’s functional capacity deteriorates, the capacity for activity (i.e. a facet of behaviour) can be improved by lowering the demands made by the environment. Moreover, according to the docility hypothesis persons with lower capacity are more sensitive to the demands of the environment than are those with higher capacity.

Widely used in, for instance, environmental gerontology and occupational therapy (see, e.g. Granbom et al. 2014; Oswald et al. 2007; Rantakokko et al. 2013), the relation between the person’s functional capacity and the demands of the housing environment is described in terms of housing accessibility (Iwarsson and Ståhl 2003). In the present study, the personal (*P*) factor concerns functional capacity in terms of functional limitations and the environmental (*E*) factor concerns design features of the housing environment in terms of physical environmental barriers (Preiser and Ostroff 2001; Steinfeld and Danford 1999). When the manifestation of functional limitations in the individual coincides with the presence of a physical environmental barrier, there is a *P* × *E* interaction. For example, if the individual has poor balance and lives in a dwelling with stairs without handrails, this interaction generates accessibility problems. In contrast, for an individual without functional limitations, stairs without handrails (i.e. a design feature not up to standard) do not generate any housing accessibility problems. That is, housing accessibility problems are generated as an interaction effect of *P* and *E*. Accordingly, in this context the *P* × *E* interaction term denotes housing accessibility.

### Housing Enabler: an instrument for housing accessibility assessment and analysis

Based on 20 years of research (Iwarsson et al. 2012), the Housing Enabler is an internationally acknowledged, reliable and valid instrument for housing accessibility assessment and analysis (Iwarsson and Slaug 2010). The Housing Enabler is available in several languages and has been adapted to the national housing standard specifications in several countries (Helle et al. 2010; Iwarsson et al. 2005). Using data collected with the Housing Enabler, the P and E components of accessibility as well as the magnitude of problems generated (i.e. *P* × *E* interaction) can be studied in depth. Since testing and optimizing reliability and validity is a continuing process, data on strengths, limitations, weaknesses and inconsistencies of the Housing Enabler have been collected systematically through the years. The Housing Enabler has been applied in research projects in Sweden and cross-nationally (see, e.g. Iwarsson et al. 2007; Lien et al. 2015), with results fed back into the instrument optimization process. Notably for the current study case, while using the Housing Enabler extensively in research we have observed that the variance of the *P* × *E* total score as an overall composite measure of accessibility problems is mostly attributed to the *P* component (Slaug et al. 2013). Consequently, the total *P* × *E* interaction score will generally be highly correlated to *P*.

### Interaction analysis in the context of person–environment fit studies

**Fig. 1:**
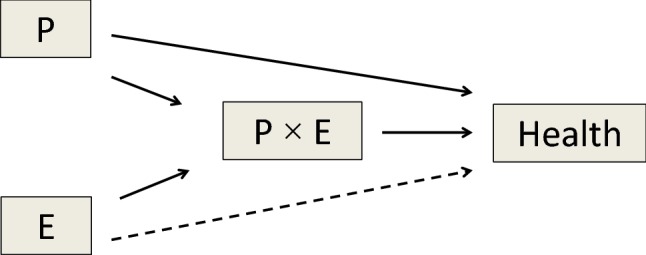
Directed Acyclic Graph (DAG) of assumed relations between explanatory variables and health outcome. In the practical application of the present paper, only minor main effect of *E* is expected on the health outcome (indicated by a dashed line in the arrow from *P* to Health), while *P* × *E* is influenced by both *P* and *E*

The assumed relationships between explanatory and outcome variables in studies using Housing Enabler data can advantageously be illustrated with a Directed Acyclic Graph (DAG; Weinberg 2007), see Fig. 1. It should be noted that in some important aspects our study case is different from settings where standard interaction analysis is applied: (a) *P* × *E* is regarded as a separate entity in the causal diagram, assumed to be caused by P (functional capacity of the person) and E (features in the built housing environment) in combination, (b) the main effect of exposure to E is expected to be minor, (c) strong correlation (multi-collinearity) between P in particular and *P* × *E* can be expected, which may inflate standard errors and thus hamper the possibility to separate the effects unless the sample size is large. These specific characteristics of studies based on Housing Enabler therefore render standard interaction analysis problematic.

To see why this is the case, we first note that in order to assess the effect of *P* × *E* on a health outcome (*Y*) without confounding in a general interaction scenario, both *P* and *E* themselves are causes that must be accounted for (see Fig. 1). A standard interaction analysis with *P*, *E* and *P* × *E* included simultaneously in a regression model would not estimate the total contribution of *P* and *E* on *Y* but only the direct effect not mediated by the *P* × *E* interaction. This is because the pathways *E*–*P* × *E*–*Y* and *P*–*P* × *E*–*Y* are blocked (i.e. taken away) by the inclusion of the interaction term *P* × *E*. In our case, adjustment for *P* is essential, whereas *E* as main effect can be omitted from the model if its independent effect is assumed to be minor. Thus, a potential solution would be to develop an analytic approach with the capacity to separate *P* × *E* interaction effects, while still keeping the main effect of *P*.

Hence, the present study targeted challenges imposed by the use in research of an instrument theoretically founded on the Ecological Theory of Ageing (Lawton and Nahemow 1973) and dealing with interactions between personal factors (*P*) and exposures to features in the built housing environment (*E*). The aim was to propose and illustrate how an analytic approach can be used to separate the *P* × *E* interaction effects from main effects of *P* and *E*, in studies where *P* and *P* × *E* are typically strongly interrelated. We used existing empirical data to investigate the association between *P* × *E* interaction and two health outcomes commonly used in research on ageing and health, that is, self-rated general health and dependence in Instrumental Activities of Daily Living (I-ADL). However, as our aim was methodological and not to answer specific empirical research questions, we intentionally reduced some of the complexity inherent in the data.

## Methods

### Data sources

Four datasets comprising Housing Enabler and health data were used. The datasets represented different populations of older people relevant for public health efforts in the context of housing. All data were collected at home visits by raters trained for reliable administration of the Housing Enabler and other instruments and questionnaires included. Details on the data collection procedures are available in the original publications referred to later on. The original studies were all approved by the Ethics Committee at Lund University or the Regional Ethical Board in Lund, Sweden, and with datasets stored in accordance with the Personal Data Act (1998:204). For the present study, only anonymized data were used.

The first dataset comprised data from the Swedish, German and Latvian national samples (*N* = 1150) of single-living very old participants from the ENABLE-AGE Project (EA; Iwarsson et al. 2007). The second dataset comprised data collected for a sub-study (Kylén et al. 2014) with a cohort from the Swedish National Study on Aging and Care (Lagergren et al. 2004) of participants randomly selected from the Swedish national population register, aged 67–70 (SNAC-GÅS; *N * = 371). The third dataset comprised data from the Home and Health in Parkinson’s Disease Project (HHPD; *N * = 255) of participants from southern Sweden of various ages with > 1-year duration of Parkinson’s disease (Nilsson and Iwarsson 2013). The fourth dataset comprised data of older persons aged 75–84 (OP; *N * = 134) from Hässleholm municipality in southern Sweden, living in ordinary housing (Iwarsson and Isacsson 1996). For sample characteristics, see Table 1.

**Table 1:** Basic characteristics of the datasets included in the present study, *N* = 1910

Characteristic	EA *n* = 1150	SNAC-GÅS *n* = 371	HHPD *n* = 255	OP *n* = 134	Total *N* = 1910
Data collection year/s	2002/2003	2010/2011	2012/2013	1994	1994–2013
Setting	Urban	Rural/semi-urban/urban	Rural/semi-urban/urban	Rural/semi-urban	Rural/semi-urban/urban
Country	Germany (*n* = 450), Latvia (*n* = 303), Sweden (*n* = 397)	Sweden	Sweden	Sweden	Germany, Latvia, Sweden
Multi-dwelling^a^, %	89.4	60.6	45.8	41.7	70.2
Sex, %					
Men	20.3	42.9	60.4	42.5	31.6
Women	79.7	57.1	39.6	57.5	68.4
Age, mean (SD)	83.4 (3.9)^b^	68.6 (0.9)	70.1 (9.3)	79.0 (2.9)	78.4 (8.0)
*P* (no of functional limitations), median (*q*1–*q*3)^c^	3 (2–5)	1 (0–2)	4 (2–6)	2 (1–3)	2 (1–4)
*E* (no of potential barriers), median (*q*1–*q*3)^c^	56 (49–63)	69 (62–76)	67 (59–74)	54 (50–59)	59 (52–68)
*P* × *E* (no of actual barriers), median (*q*1–*q*3)^c^	20 (9–31)	0 (0–12)	28 (18–36)	10 (0–20)	17 (4–29)

EA, ENABLE-AGE (Iwarsson et al. 2007); SNAC-GÅS, sub-study of the Swedish National Study on Aging and Care (Kylén et al. 2014); HHPD, Home and Health in Parkinson’s Disease (Nilsson and Iwarsson 2013); OP, Older Persons from Hässleholm municipality in southern Sweden, living in ordinary housing (Iwarsson and Isacsson 1996)

^a^Term used in Sweden to denote housing with multiple apartments

^b^Different age spans in Germany and Sweden (80–89 years) and Latvia (75–84 years)

^c^*q*1–*q*3: inter-quartile range

### Housing Enabler: accessibility problems

Accessibility problems were assessed with the Housing Enabler instrument (Iwarsson and Slaug 2010), which is administered in three steps (see, e.g. Iwarsson et al. 2012). Step 1 (*P* component) concerns 12 items of functional limitations (such as visual impairment, loss of hearing, poor balance, incoordination, limitations of stamina, etc.) and two items on dependence on mobility devices (treated as a proxy for more severe functional limitations), rated as present/not present in a person, based on an interview conducted in combination with professional observation. Step 2 (*E* component) consists of observation of the physical housing environment, with a detailed rating of the occurrence of 161 potential environmental barriers in the exterior surroundings (28 items), in the entrance (46 items) and in the indoor environment (87 items), rated as present/not present based on national guidelines and standards for housing design (i.e. specifications for the design of specific housing features). Importantly, the E component is administered based on observations of design features as they appear at the time of the assessment, no matter if they were part of the original design of the dwelling or the result of individual housing adaptations or refurbishment. The environmental and personal components are objectively assessed independently of each other, and no information on the individual’s perceptions of barriers is included. In the analyses, P and E are two discrete indices representing the number of functional limitations and potential environmental barriers present, respectively. Step 3 (*P* × *E* interaction) is based on the assessments in steps 1 and 2, and *P* × *E* scores are computed by means of a matrix procedure which juxtaposes the items of P with the items of E (for details, see Iwarsson et al. 2012).

For this study, in order to more straightforwardly address the methodological issue at target, we used a simplified scoring procedure applying a dichotomization (0 = no score generation, 1 = score generation) instead of a graded score (as stipulated by the original instrument) to compute the *P* × *E* interaction scores. Moreover, only the environmental barriers generating the most severe accessibility problems were included. Thus, in the current study the *P* × *E* score represents the number of barriers actually generating severe/impossible accessibility problems in each individual case; we will henceforth refer to these as *actual barriers*. In contrast, the *E* component represents *potential barriers*, that is all barriers assessed as present in the housing environment, regardless if they generate accessibility problems in the individual case or not.

### SF-36: self-rated general health

To capture self-rated general health, the question “In general would you say your health is?” from the SF-36 questionnaire was used (Ware and Sherbourne 1992), with general health rated on a scale with five response alternatives ranging from “Poor” (1) to “Excellent” (5). All four datasets contained data collected with this question, but for consistency across the datasets we transposed a 7 grade scale used in the OP dataset to the 5 grade scale used in the other datasets. For the multi-variable logistic regression analyses, self-rated health was dichotomized into 0 = Poor/Fair and 1 = Good/Very good/Excellent.

### ADL staircase: dependence in I-ADL

We used data collected with the four I-ADL items (i.e. cooking, shopping, cleaning and transportation) of the ADL Staircase (Sonn and Hulter-Åsberg 1991). Data were collected using the three-graded scale, independent/partly dependent/dependent. Following instructions of the instrument manual, the partly dependent category was recoded to independent in one instance (cleaning) and to dependent for the remaining three items. For the multi-variable regression analyses, we created a dichotomized I-ADL variable, where 0 = independent in all four activities, and 1 = dependent in at least one activity.

### Statistical analysis

In order to separate the *P* × *E* interaction effect from the main effects of *P* and *E*, we developed an analytic approach in two distinct steps.

In a first step, linear regression was conducted with the natural logarithm of *P* as the independent variable and *P* × *E* (i.e. computed based on the actual barriers of the *E* component) as the dependent variable. The natural logarithm of *P* was used to improve model fit. Individuals with *P* = 0 were not included in this first step, as these individuals have zero actual barriers (*P* × *E* = 0) by definition and would thus not contribute to the predictions. The amount of residual variability present in this first step indicates to what extent it will be statistically possible to separate the interaction effect *P* × *E* from the main effect of P. The linear regression was carried out (and graphically displayed) on the four datasets separately, and the residuals were kept in each dataset as a measure of how many more (or fewer) actual barriers than expected (given the number of functional limitations and the *P* × *E* scores they generate) each person had in his/her housing environment. A positive residual thus implies that the person lived in a housing environment with more actual barriers than expected, whereas a negative residual represents a case with fewer actual barriers than expected. Accordingly, the independent variable *P* and the resulting *P* × *E* residuals will be uncorrelated under standard assumptions of linear regression analysis. The *P* × *E* residuals were divided into the categories: (1) same (or about the same) number of actual barriers as expected, (2) fewer actual barriers than expected and (3) more actual barriers than expected. As cut-off for this categorization we chose a difference from the expected number of actual barriers in any direction that gave a fairly even distribution between the three categories, namely at least four barriers more than or less than expected. As an additional category enumerated 0, we accounted for the cases where the number of actual barriers (i.e. those barriers that generate accessibility problems in relation to certain functional limitations in the individual case) was 0 due to the absence of functional limitations. The distribution of categorized *P* × *E* residuals was examined in the four datasets separately. For the subsequent analyses, the four datasets were pooled into one (*N * = 1910).

In a second step, we conducted multi-variable regression analysis with P and the *P* × *E* residuals categorized 0–3 as described above as explanatory variables, and the health outcome under investigation (i.e. self-rated health or I-ADL dependence, respectively) as the dependent variable. In the present study case, we employed logistic regression, but it could be linear or ordinal depending on the scale characteristics of the dependent variable under study. Standard adjustment for other individual-level factors we considered of importance for the investigated health outcome was included: age, sex and dataset by country (six categories). The *p* values obtained for the *P* × *E* residuals in the regression models can be used to assess the evidence for a *P* × *E* interaction effect, while controlling for the influence of P. The parameter estimates associated with the categorized *P* × *E* residuals represent the effect of living in a more or less accessible housing environment (operationalized as actual barriers) than expected, estimated as though the number of functional limitations (*P*) was the same across these four categories.

In addition, we conducted a comparison between standard single-step interaction analysis and the proposed two-step approach, using continuous interaction terms (i.e. *P* × *E* vs. *P* × *E* residuals) in both models and with the same adjustment variables.

All computations were conducted using IBM SPSS Statistics 22 for Windows (IBM Corporation, Armonk, NY, U.S.). *p* values < 0.05 were considered statistically significant.

## Results

**Fig. 2:**
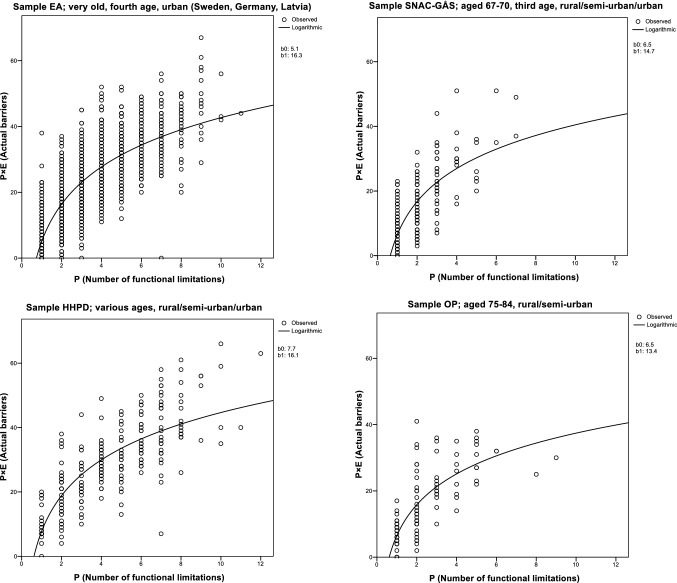
The association between P (number of functional limitations) and P × E (number of environmental barriers generating severe/impossible accessibility problems, i.e. actual barriers) in four datasets, analysed by means of linear regression. The association was strong (*r* ≥ 0.80) and statistically significant (*p* < 0.001) in all four datasets

The results of the first step of our analytic approach, that is, the linear regression analysis with the natural logarithm of *P* (number of functional limitations) and *P* × *E* (number of actual barriers) in the four datasets, are shown in Fig. 2. Across the four datasets, the association was similarly strong (*r* ≥  0.80) and highly significant (*p* < 0.001), with a sharp increase in the number of actual barriers displayed starting already for cases with 1–2 functional limitations. With higher numbers of functional limitations, the apparent effect of increased number of actual barriers levelled out. The residual variability of the regression model in Fig. 2 indicated that it would be statistically possible to separate the interaction effect *P* × *E* from the main effect of *P*.

The distribution of the categorized *P* × *E* residuals differed somewhat across the datasets, mainly in terms of the proportion where no barriers generated accessibility problems due to absence of functional limitations (49.6% in the SNAC-GÅS dataset, compared to 8.0, 5.9 and 20.9%, respectively, in the others). For detailed information, see Table 2.

**Table 2:** Distribution of *P* × *E* residuals across the four datasets

	EA *n* = 1150	SNAC-GÅS *n* = 371	HHPD *n* = 255	OP *n* = 134	Total *N * = 1910
	Column %				
Category of *P* × *E* residuals					
(0) No barriers generating problems, due to no functional limitations	8.0	49.6	5.9	20.9	16.7
(1) Expected number of actual barriers	40.3	21.6	38.4	38.1	36.3
(2) Fewer actual barriers than expected	28.1	15.6	29.0	23.1	25.4
(3) More actual barriers than expected	23.6	13.2	26.7	17.9	21.6

EA, ENABLE-AGE (Iwarsson et al. 2007); SNAC-GÅS, sub-study of the Swedish National Study on Aging and Care (Kylén et al. 2014); HHPD, Home and Health in Parkinson’s Disease (Nilsson and Iwarsson 2013); OP, Older Persons from Hässleholm municipality in southern Sweden, living in ordinary housing (Iwarsson and Isacsson 1996)

The distributions of self-rated general health and dependence in daily activities in relation to the categorized *P* × *E* residuals are shown for the pooled dataset in Table 3. Notably, the participants with no functional limitations rated their general health higher and were more independent compared to those included in categories 2 and 3.

**Table 3:** Self-rated general health^a^ and dependence in I-ADL^b^ in relation to categories of *P* × *E* residuals; pooled dataset, *N * = 1910

	Self-rated general health					I-ADL				
	“In general would you say your health is?”					Number of activities performed independently				
	Poor	Fair	Good	Very good	Excellent	0	1	2	3	4
	Row %					Row %				
Category of *P* × *E* residuals										
(0) No barriers generating problems, due to no functional limitations	0.0	7.8	28.2	36.4	27.6	0.9	0.3	6.3	11.4	81.1
(1) Expected number of actual barriers	9.0	39.9	29.2	16.6	5.2	6.2	9.1	11.9	22.6	50.4
(2) Fewer actual barriers than expected	12.6	41.5	28.3	13.0	4.5	8.7	8.3	13.1	16.9	53.0
(3) More actual barriers than expected	10.0	44.1	28.0	11.7	6.1	9.8	12.5	16.3	21.8	39.6

^a^Item from the SF-36 Questionnaire (Ware and Sherbourne 1992)

^b^I-ADL, Instrumental Activities of Daily Living (Sonn and Hulter-Åsberg 1991)

In the second step of our analytic approach, the regression analysis indicated a significant association (*p* < 0.001) between the categorized *P* × *E* residuals and self-rated general health. The direction of the odds ratio suggested that more actual barriers were associated with less likelihood of rating good health (OR = 0.72, 95% CI 0.54–0.95). The logistic regression with dependence in I-ADL as the dependent variable did not provide clear evidence of an association (*p* = 0.275) between more actual barriers than expected and being dependent in at least one activity. The number of functional limitations (*P*) was significantly associated with both health outcomes (self-rated general health, OR = 0.74, 95% CI 0.69–0.79; I-ADL, OR = 1.47, 95% CI 1.37–1.58). For further details, see Table 4.

**Table 4:** Multi-variable logistic regression analysis, with self-rated general health^a^ (0 = Poor/Fair, 1 = Good/Very good/Excellent) and dependence in I-ADL^b^ (0 = independent in all four activities, 1 = dependent in at least one activity) as dependent variables; pooled dataset *N * = 1910

	Self-rated general health				I-ADL			
	*B*	OR	95% CI	*p* value	*B*	OR	95% CI	*p* value
P (Number of functional limitations)	− 0.31	0.74	0.69–0.79	**< 0.001**	0.39	1.47	1.37–1.58	**< 0.001**
Category of *P* × *E* residuals				**< 0.001**				0.275
(0) No barriers generating problems, due to no functional limitations	1.26	3.53	2.12–5.87	**< 0.001**	0.13	1.14	0.75–1.72	0.549
(1) Expected number of actual barriers	0.0	1.0	Ref.		0.0	1.0	Ref.	
(2) Fewer actual barriers than expected	0.02	1.02	0.77–1.35	0.893	− 0.14	0.87	0.66–1.15	0.337
(3) More actual barriers than expected	− 0.34	0.72	0.54–0.95	**0.023**	0.27	1.18	0.88–1.60	0.271
Confounders: age	0.00	1.00	0.97–1.02	0.852	0.09	1.10	1.07–1.13	**< 0.001**
Sex (female)	− 0.38	0.68	0.53–0.88	**0.004**	− 0.59	0.55	0.43–0.72	**< 0.001**
Dataset and country				**< 0.001**				**< 0.001**
Dataset and country, EA Sweden	0.0	1.0	Ref.		0.0	1.0	Ref.	
Dataset and country, EA Germany	− 1.54	0.22	0.16–0.30	**< 0.001**	0.18	1.20	0.88–1.64	0.249
Dataset and country, EA Latvia	− 3.15	0.04	0.03–0.07	**< 0.001**	− 0.94	0.39	0.26–0.57	**< 0.001**
Dataset and country, SNAC-GÅS Sweden	− 0.40	0.67	0.38–1.18	0.163	− 0.71	0.49	0.27–0.91	**0.024**
Dataset and country, HHPD Sweden	− 0.46	0.63	0.37–1.07	0.089	0.52	1.68	0.94–3.02	0.082
Dataset and country, OP Sweden	− 0.93	0.39	0.24–0.64	**< 0.001**	1.30	3.69	2.29–5.95	**< 0.001**
Nagelkerke *R* square	0.431				0.409			

Significant *p* values (< 0.05) are bolded

^a^ Item from the SF-36 Questionnaire (Ware and Sherbourne, 1992) ^b^ I-ADL, Instrumental Activities of Daily Living (Sonn and Hulter-Åsberg, 1991)

More pronounced main effects of the P component expressed as odds ratios were observed under the new two-step approach compared to the standard single-step approach, which is due to differences in the way the models are parameterized. The new approach estimates the total effect of P, whereas the standard approach only estimates the direct effect not mediated by *P* × *E* interaction (see Table 5). The standard and new approach yielded similar and clear evidence for *P* × *E* interaction for both health outcomes.

**Table 5:** Standard single-step approach of testing interaction effects versus proposed two-step approach

	Self-rated general health				I-ADL			
	*B*	OR	95% CI	*p* value	*B*	OR	95% CI	*p* value
Standard single-step approach								
*P* (number of functional limitations)	− 0.24	0.79	0.72–0.87	**< 0.001**	0.27	1.31	1.14–1.45	**< 0.001**
*P* × *E* (actual barriers)	− 0.03	0.97	0.96–0.99	**< 0.001**	0.02	1.02	1.00–1.04	**0.010**
Nagelkerke *R* square	0.420				0.410			
Proposed two-step approach								
*P* (number of functional limitations)	− 0.40	0.67	0.63–0.72	**< 0.001**	0.38	1.46	1.37–1.55	**< 0.001**
*P* × *E* residuals (categories 0–3)	− 0.02	0.98	0.97–1.00	**0.026**	0.03	1.03	1.01–1.04	**0.002**
Nagelkerke *R* square	0.414				0.412			

Significant *p* values (< 0.05) are bolded

^a^Item from the SF-36 Questionnaire (Ware and Sherbourne 1992)

^b^I-ADL, Instrumental Activities of Daily Living (Sonn and Hulter-Åsberg 1991)

## Discussion

The main result of the present study is that the analytic approach proposed was shown to have the potential of increasing the possibilities for valid tests of research questions based on theoretical assumptions such as the docility hypothesis. Using a well-documented study case in order to test and illustrate the suggested approach, the interaction effects of *P* × *E* were separated from the main effect of *P* in regression models with two different health outcomes, that is, self-rated general health and dependence in I-ADL. The regression analyses indicate that more actual barriers in the housing environment than expected from the number of functional limitations are negatively associated with self-rated general health (Table 4). The results regarding I-ADL were less consistent since an interaction was noted only when the *P* × *E* residuals were used as a continuous explanatory variable and not when the residuals were categorized. However, in both models the direction of the odds ratios was such that more barriers in the housing environment than expected from their functional limitations increased the risk for dependence in I-ADL and vice versa. A common interpretation of a significant interaction effect in a model with main effects of both person and environment included is that the combined person–environment effect is different from summing the person and environment contributions separately in an additive model or different from multiplying the person and environment contributions separately in a multiplicative model. Our analyses however were based on the assumption that environmental main effect is marginal. The results suggest that the housing environment modifies the association between functional limitations and the health outcomes studied, such that decreased functional capacity has more profound consequences than could be expected from a multiplicative model in the presence of barriers. This finding can also be meaningfully interpreted as showing that those with lower functional capacity are more sensitive to environmental demands, which is in line with the theoretical foundation of our study case (the Ecological Theory of Ageing; see, e.g. Oswald et al. 2007).

There have been earlier attempts to explore the assumption that there are *P* × *E* interaction effects on health outcomes in the context of housing accessibility, going beyond a multiplicative model. To date, several alternative analytic approaches have been pursued, such as the utility of an item-specific *P* × *E* function (Granbom et al. 2014) and an environmental barrier rank-order approach computed using the *P* × *E* function (Pettersson et al. 2015; Rantakokko et al. 2013). However, these approaches identified further threats to the validity of the findings. In Granbom et al. (2014), the item-specific *P* × *E* function was used to compare the extent to which each environmental barrier (of a checklist comprising 61 different barriers) generated problems among very old people (80–89 years old) before and after moving. It was an attempt to pinpoint accessibility problems at a detailed level, but the large number of statistical testing involved implies the risk of mass significance. In the studies by Pettersson et al. (2015) and Rantakokko et al. (2013), the rank-order approach was used to identify the most problematic environmental barriers based on the sample-specific prevalence of functional limitations in relation to the occurrence of environmental barriers. The environmental barriers generating most problems were then used in analyses of mortality risks. The item-specific exposure effects were thus not specific to each person’s particular functional status and abilities. Accordingly, some of those environmental barriers identified as important at the group level may not be relevant to all individuals in a sample, thus rendering the usual average interpretation of exposure effects estimated from regression analyses less meaningful (Pettersson et al. 2015; Rantakokko et al. 2013). Against this background, the new approach, that is, creating a variable that can be used in regression models together with P while still validly representing the *P* × *E* interaction, emerges as more promising and with the potential to contribute to the much needed deepening of the knowledge in this field of inquiry.

The importance of the initial step of regressing *P* × *E* against *P* needs to be stressed. That is, the variability of the *P* × *E* residuals will—together with the proportion of individuals with residuals above and below expectation—indicate to what extent it will be statistically possible to separate the interaction effect *P* × *E* from the main effect of *P*. Thus, the first step paves the way for the second step where health outcomes can be regressed against P and the *P* × *E* residuals as two essentially independent constructs, as demonstrated by our study case. Accordingly, on condition of passing the initial step, our two-step approach comprehensibly separates the *P* × *E* interaction effect from the effect of *P* which was a primary objective of the present study. The diversity of settings and contexts of the datasets successfully used in our study case gives reason to be positive about the feasibility of applying this new approach in future studies.

Moreover, the similar patterns of association between functional limitations and number of actual barriers displayed in the four datasets are striking (see Fig. 2), considering that the datasets represent different countries, age groups and even diagnostic status (one sample comprising only persons with diagnosed Parkinson’s disease). While we did not aim to answer any empirical research questions in our study, this was essential for the development of our analytic approach, as we could explore and test it across the different datasets with comparable results, and also pool the datasets for the illustration with health outcomes. For research questions focusing on testing theoretical assumptions and theory driven hypotheses, the simplified scoring procedure used in the current study could be advisable. However, the reduction in variance in data that follow from only counting the number of barriers generating the most severe problems makes the simplified scoring procedure less appropriate for questions searching for explanations at the individual level. For such research questions, using the original scoring procedure of the Housing Enabler is still recommended. However, the approach can also be applied with the original scoring procedure, just replacing actual barriers with the original total accessibility score in the first analytic step. It should also be noted that even though we chose to categorize the residuals in our study case illustration, the residuals can just as well be used in the second step as a continuous variable, depending on the research question, the anticipated association with the health outcome and scale properties of other variables in the regression models.

The specifics of the construct used in the Housing Enabler to capture certain person–environment fit issues (a complex matrix procedure that render some combinations of P and E problematic and others not, see, e.g. Carlsson et al. 2009; Iwarsson et al. 2012) may give the impression that our proposed approach has a limited scope. The notion of interaction effects caused by the contributing factors *P* and *E* is, however, not new or restricted to this scope of research. Failure to control for *P* and *E* yields confounded results regarding the presence of interaction. In other areas of epidemiological research, the interaction term is usually referred to as cumulative exposure and is calculated as the result of multiplication of average intensity and duration of exposure (de Vocht et al. 2015). That could be seen as a parallel to our study case, where the interaction term can be considered as a cumulative exposure metric, resulting from the degree of reduced functional capacity and the amount of barrier exposure. Our methodological contribution highlights the importance of a careful interpretation of a standard interaction analysis with main effects (exposure intensity and duration) and an interaction effect (cumulative exposure) included in the regression model simultaneously. Contrary to our second-stage model, the main effect variables of a standard interaction model do not yield estimates of the total contribution of exposure intensity and duration on health outcomes, since part of the effect is blocked by inclusion of the cumulative exposure metric calculated from intensity and duration. Neither exposure intensity nor duration can generally be ignored (de Vocht et al. 2015), and the procedure we propose can therefore not be applied in other fields of research without further extension. That is, even though our approach in itself is not restricted to a certain field of research, the procedure developed was tailored to target specific challenges imposed by the use in research of an instrument (Housing Enabler) dealing with the relation between the person’s functional capacity and the demands of the housing environment. Thus, applying the analytic approach outlined in this study to data collected with other instruments requires further methodological examination in order to validly account for intensity and duration of exposure.

### Strengths and limitations of the study

A strength of the present study is that we had access to a rich data source that represents a variety of settings and contexts. The results were strikingly consistent across the four datasets, which is an indication of the validity of our proposed analytic approach. A main limitation is the cross-sectional study design, meaning that changes in the housing environment as a result of the health status cannot be ruled out.

## Conclusions

The two-step—instead of single-step—analytic approach proposed for investigating person–environment interactions in studies of housing accessibility and health emerged as promising in order to separate the interaction effect in multi-variable regression models. The new approach has the potential to increase the possibilities for sound and meaningful tests of underlying theoretical assumptions.
